# Giant Cell Tumour of the Tendon Sheath of the Hand: A Retrospective Review With Recurrence Risk Analysis

**DOI:** 10.7759/cureus.91159

**Published:** 2025-08-28

**Authors:** Pedro Balau, Manuel Magalhães Godinho, Marta Santos Silva, Vera Resende, Artur Costa Neto

**Affiliations:** 1 Orthopaedics and Traumatology, Unidade Local de Saúde de Entre o Douro e Vouga, Santa Maria da Feira, PRT

**Keywords:** giant cell tumour, hand, recurrence, tendon sheath, tumour

## Abstract

Introduction: Giant cell tumours of the tendon sheath (GCT-TS) are a benign but potentially recurrent soft tissue tumour of the hand. It typically presents as a slow-growing, painless mass and is most frequently located on the volar aspect of the fingers. Despite its benign nature, recurrence rates vary widely across published series.

Methods: This retrospective study analysed 90 histologically confirmed cases of GCT-TS treated surgically between 2011 and 2023 in a single orthopaedic surgery department. Demographic, clinical, imaging, and histological data were collected. A phone survey was conducted to assess long-term outcomes and possible recurrence. In cases of suspected recurrence, in-person evaluation was offered.

Results: A total of 85 patients (51 female, 34 male) were included, with a mean age of 50 years. The index finger was the most commonly affected location (31.8%), and the average lesion size was 17.6 mm. No atypical histological features were found. The recurrence rate confirmed by histology was 5.6% (n = 5), increasing to 10% (n = 9) when including clinically suspected cases. Statistical analysis revealed a significant association between recurrence and lesion location, with all recurrences occurring in finger joints (p = 0.013). Multivariable analysis confirmed joint-related location as an independent risk factor (odds ratio (OR) = 0.09; 95% confidence interval (CI): 0.01-0.80; p = 0.026). Post-operative complications were minimal, and patient satisfaction was high.

Conclusion: Our findings show a recurrence rate at the lower end of published data. Lesions involving finger joints were significantly more likely to recur. Pre-operative imaging and meticulous surgical technique are crucial to minimise recurrence and optimise outcomes.

## Introduction

Giant cell tumours of the tendon sheath (GCT-TS) are among the most common hand tumours, usually presenting as a firm, nodular and painless slow-growing mass of the volar aspect of the hand and fingers. The most common locations in the hand are the second and third fingers, near the distal interphalangeal joint. It is most commonly found in women and can occur at any age, with peak incidences in the third to fourth decades [1].

The most usual and accepted treatment is complete local excision, with some reports of local radiotherapy [2], although its role is yet to be clearly defined [1,3]. Despite its benign nature, local recurrence after surgical excision is reported as high as 44% [4], but more recent studies reported lower recurrences [3,5,6]. There are no proven and widely accepted risk factors for recurrence, except for incomplete excision [1,3].

This review aims to present our experience with GCT-TS of the hand. We conducted a retrospective analysis of all cases operated in our department between 2011 and 2023, focusing on demographic, clinical, and histological characteristics, and comparing our recurrence rate with those reported in the literature. Given the variability of recurrence rates and the limited statistical validation of potential prognostic factors in prior series, our study also sought to explore whether specific clinical or anatomical features could be identified as predictors of recurrence.

## Materials and methods

A retrospective review was performed of all cases treated in our department with a confirmed diagnosis of GCT-TS by histologic analysis between 2011 and 2023. We reviewed clinical and operative records, pathology reports, and available imaging studies, such as plain radiographs, ultrasound (US), and magnetic resonance imaging (MRI). Demographic data, including gender and patient age at the time of surgery, were collected, along with the anatomical location of the lesion. Pathology reports were analysed to confirm the diagnosis, assess the macroscopic dimensions of the lesion, and evaluate histopathological features. Imaging studies were reviewed to identify abnormalities potentially associated with recurrence, such as joint invasion or bone erosion. A phone survey was conducted to assess post-operative outcomes. This included evaluation of residual symptoms and functional impairment, local clinical recurrence, and overall patient satisfaction with the procedure. In cases where doubts persisted regarding recurrence or post-operative complications, the patient was asked to come to the outpatient clinic for further evaluation.

Patient characteristics were summarised as mean and standard deviation (SD) or median and interquartile range (IQR) for continuous variables and absolute and relative frequency for categorical variables. Groups were compared by recurrence status using Welch’s t‑test or the Mann-Whitney U test for continuous variables, and the χ^2^ test or Fisher’s exact test for categorical variables, as appropriate. For 2×2 tables with a zero cell, we applied the Haldane-Anscombe correction (adding 0.5 to each cell) to compute crude odds ratios (ORs) and 95% confidence intervals (CIs). Two‑sided p‑values < 0.05 were considered statistically significant. Univariable and multivariable logistic regression models were fitted with recurrence as the outcome. The multivariable model included sex, age at surgery, lesion location, and lesion size. Models were estimated by maximum likelihood. When complete separation was present, we used Firth’s bias‑reduced penalised likelihood, reporting penalised ORs with profile‑likelihood 95% CIs. Analyses were performed in R Statistical Software (v4.3.3; R Foundation for Statistical Computing, Vienna, Austria) and Firth’s bias-reduced penalised likelihood was implemented through the logistf R package (v1.26.1; Heinze G, Ploner M, Jiricka L, Steiner G, 2025).

## Results

Between 2011 and 2023, we treated 85 patients with a total of 90 histologically confirmed cases of GCT-TS of the hand. Of the 85 patients included in the study, 51 were female (60%) and 34 were male (40%). The mean age at the time of surgery was 50.1 years (SD ± 13.6, range 15-88).

The most commonly affected location was the index finger, accounting for 31.8% of cases (n = 27), followed by the long finger with 17.6% (n = 15), the thumb with 15.3% (n = 13), the ring finger with 14.1% (n = 12), the palm of the hand with 11.8% (n = 10), and the little finger with 9.4% (n = 8). The right hand was involved in 56.5% of cases (n = 48), while the left hand was affected in 43.5% (n =37).

The average lesion size was 17.6 mm, with a median of 15 mm (IQR 5-20), ranging from 5 mm, observed in a case involving the ring finger, to a maximum of 50 mm in a thumb lesion. No atypical features were identified in the pathology reports. On microscopic examination, all tumours were characterised by the presence of multinucleated giant cells and a diffuse fibrohistiocytic proliferation.

We were able to access imaging reports for 55% of the patients included in the study. Among these, 21 patients underwent MRI, while 26 patients had US performed. For the remaining patients, either the imaging reports originated from external institutions and were not retrievable, or no imaging documentation was available in our clinical information system at the time of data collection. In the subset of patients for whom imaging was accessible, the reports indicated the absence of intra-osseous involvement or intra-articular extension of the lesions. Additionally, no signs of osteoarthritic degeneration or joint space narrowing were noted in any of the available imaging studies.

The phone survey was successfully conducted in 84 out of 85 patients, as one patient was deceased at the time of follow-up. The final follow-up duration ranged from 15 to 169 months. Regarding post-operative outcomes, one patient (n = 1) reported persistent decreased distal sensation in the affected finger. Three patients (n = 3) described a mild hypersensitive surgical scar, and one patient (n = 1) reported joint stiffness with no impact on daily activities. There were no documented cases of post-operative infection or other surgical complications.

A total of 90 histologically confirmed GCT-TS were surgically treated in 85 unique patients. Among these, one patient experienced two recurrences, while three other patients had a single recurrence each. Following the phone survey, four patients were clinically suspected of having a recurrence and were subsequently evaluated in person. However, none of these patients chose to undergo revision surgery, and thus, histological confirmation was not obtained in these cases. The recurrence cases are illustrated in Table 1.

**Table 1:** Recurrence cases. PIP: proximal interphalangeal joint; DIP: distal interphalangeal joint; mm: millimetres

Patient ID	Gender	Lesion location		Age at the time of the surgery	Lesion size (mm)	Type of recurrence
1	Female	Ring finger	PIP	45	20	Histologically confirmed
				48	17	Histologically confirmed
				50	5	Histologically confirmed
2	Female	Ring finger	DIP	48	10	Histologically confirmed
				52	15	Histologically confirmed
3	Female	Long finger	DIP	51	15	Histologically confirmed
				54	6	Histologically confirmed
4	Male	Little finger	PIP	33	25	Histologically confirmed
				36	20	Histologically confirmed
5	Female	Little finger	PIP	29	10	Clinical recurrence
6	Female	Index finger	DIP	63	20	Clinical recurrence
7	Female	Little finger	DIP	75	15	Clinical recurrence
8	Female	Index finger	DIP	57	15	Clinical recurrence

Based on confirmed histopathology, the recurrence rate was 5.6% (n = 5 out of 90 lesions). When including both histologically confirmed and clinically suspected cases, the total recurrence rate in our series was 10% (n = 9).

Statistical analysis (Table 2) revealed a significant association between tumour location and recurrence. Lesions located in the finger joints were associated with a higher recurrence rate than those in other anatomical locations (100% vs. 61.7%, p = 0.013). Female sex showed a non-significant trend toward higher recurrence (88.9% of recurrence cases vs. 58.0% in non-recurrence; p = 0.146). There were no significant differences in age (p = 0.975) or lesion size (p = 0.887) between recurrence and non-recurrence groups.

**Table 2:** Characteristics of the sample and comparison with recurrence. * Two‑sided p‑values < 0.05 were considered statistically significant. In "gender" and "lesion location," Fisher’s exact test was used. In "age," Welch’s t‑test was used. In "lesion size," Mann-Whitney's U test was used. SD: standard deviation; IQR: interquartile range; IP: interphalangeal joint; DIP: distal interphalangeal joint; PIP: proximal interphalangeal joint; MCP: metacarpophalangeal joint

		Overall (N = 90), N (%)	No recurrence (N = 81), N (%)	Recurrence (N = 9), N (%)	P-value
Gender: female		55 (61.1)	47 (58.0)	8 (88.9)	0.146
Age, mean (SD)		50.1 (13.6)	50.1 (13.6)	50.3 (14.0)	0.975
Lesion location	IP/DIP	35 (38.9)	30 (37.0)	5 (55.6)	0.270
	Middle phalanx	9 (10.0)	9 (11.1)	0 (0.0)	
	PIP	19 (21.1)	15 (18.5)	4 (44.4)	
	Proximal phalanx	12 (13.3)	12 (14.8)	0 (0.0)	
	MCP	5 (5.6)	5 (6.2)	0 (0.0)	
	Metacarpal/wrist	10 (11.1)	10 (12.3)	0 (0.0)	
Lesion size (mm), median (IQR)		15 (5, 20)	15 (11, 20)	15 (15, 20)	0.887
		Overall (N = 90)	No recurrence (N = 81)	Recurrence (N = 9)	P-value
Gender: female		55 (61.1)	47 (58.0)	8 (88.9)	0.146
Age, mean (SD)		50.1 (13.6)	50.1 (13.6)	50.3 (14.0)	0.975
Lesion location	Finger joints	59 (65.6)	50 (61.7)	9 (100)	0.013*
	Other locations	31 (34.4)	31 (38.3)	0 (0.0)	
Lesion size (mm), median (IQR)		15 (5, 20)	15 (11, 20)	15 (15, 20)	0.887

In the multivariable logistic regression model (Table 3), female sex (OR = 3.30; 95% CI: 0.63-33.94; p = 0.170), age (OR = 0.99; 95% CI: 0.93-1.05; p = 0.725) and lesion size (OR = 1.00; 95% CI: 0.89-1.10; p = 0.966) were not significant predictors.

**Table 3:** Results of the univariable and multivariable models for recurrence prediction. * Two‑sided p‑values < 0.05 were considered statistically significant. OR: odds ratio; 95% CI: 95% confidence interval; IP: interphalangeal joint; DIP: distal interphalangeal joint; PIP: proximal interphalangeal joint; MCP: metacarpophalangeal joint

		Univariable analysis		Multivariable analysis	
		OR (95% CI)	P-value	OR (95% CI)	P-value
Gender: female		4.12 (0.86-39.94)	0.078	3.26 (0.64-32.6)	0.165
Age		1.00 (0.95-1.05)	0.976	0.99 (0.93-1.06)	0.773
Lesion location	IP/DIP	(reference)			
	Middle phalanx	0.29 (0.01-2.99)	0.450	0.29 (0.01-3.22)	0.363
	PIP	1.61 (0.38-6.53)	0.504	1.52 (0.33-7.03)	0.584
	Proximal phalanx	0.22 (0.01-2.21)	0.234	0.21 (0.01-2.31)	0.233
	MCP	0.50 (0.01-5.65)	0.633	0.50 (0.01-6.68)	0.649
	Metacarpal/wrist	0.26 (0.01-2.67)	0.305	0.33 (0.01-4.08)	0.444
Lesion size (mm)		0.98 (0.88-1.07)	0.738	1.00 (0.89-1.11)	0.980
		Univariable analysis		Multivariable analysis	
		OR (95%CI)	p-value	OR (95%CI)	p-value
Gender: female		4.12 (0.86-39.94)	0.078	3.30 (0.63-33.94)	0.170
Age		1.00 (0.95-1.05)	0.976	0.99 (0.93-1.05)	0.725
Lesion location	Finger joints	(reference)			
	Other locations	0.08 (0.01-0,71)	0.017*	0.09 (0.01-0.80)	0.026*
Lesion size (mm)		0.98 (0.88-1.07)	0.738	1.00 (0.89-1.10)	0.966

Lesion location outside of the finger joints was associated with a lower recurrence risk (OR = 0.08; 95% CI: 0.01-0.71; p = 0.017). The odds of recurrence in the finger joints are estimated to be 12.5 times higher than in other locations. This difference remained statistically significant after adjusting for gender, age, and lesion size (OR = 0.09; 95% CI: 0.01-0.80; p = 0.026).

## Discussion

Patients with GCT-TS usually present a palpable mass on their hands, either with no symptoms or with limited range of motion or pain, due to proximity to the finger joints [1]. After complementary imaging, excision can be offered to the patient to relieve symptoms and to clarify the diagnosis. US is a useful tool that provides valuable diagnostic information in these lesions. It can differentiate between solid and cystic tumours and can identify potential satellite lesions. In addition, it can also provide information about its location in relation to other structures [7]. Simple radiographs can also be requested to evaluate bone erosion or invasion and to diagnose arthritic joints. Magnetic resonance can provide a more detailed insight into what tissues are involved and their spatial location, for more detailed surgical planning [7-9].

Regarding the demographics found in our series, it goes along with other published series, where women are more affected by men (60% vs. 40% respectively), and the mean age is within the normal range. Fotiadis et al., in their 2011 systematic review, reported a male-to-female ratio of 1:1.47, the same as our series [1]. Tumour location is also in line with the results of this systematic review, where it reports the index finger being the most affected in 29.7% (vs. 31.8% in our series). We had a slightly higher percentage of cases in the palm and a slightly lower percentage in the little finger.

These tumours are characterised by a relatively high rate of local recurrence, with recurrence rates reported in the literature being highly variable. Reports describe rates ranging from 4% to as high as 44%; however, more recent studies suggest that the average recurrence rate is lower [3,4,6]. Our overall recurrence rate was 10%, including both histologically confirmed and clinically suspected cases. This value remains at the lower end of published data. Among the recurrence group, 88.9% were female, compared to 58.0% in the non-recurrence group, although this difference was not statistically significant (p = 0.146). Age and lesion size also showed no association with recurrence. Recurrences occurred exclusively in cases where the finger joints were involved. This association was statistically significant (p = 0.013) and further supported by multivariable logistic regression, where non-articular locations were associated with a significantly lower odds of recurrence (OR = 0.09; 95% CI: 0.01-0.80; p = 0.026). This suggests that joint involvement may play a role in surgical difficulty and residual tumour risk.

Rao and Vigorita described an increase in both mitotic activity and cellularity in cases of recurrent lesions. However, despite these findings, they did not establish a clear correlation between these features and the recurrence rate [10]. Al-Qattan and Kitagawa et al. did not consider them to be reliable prognostic indicators for recurrence [11,12].

Al-Qattan proposed a novel classification system for these tumours, categorising them into two types. Type I tumours are defined as solitary lesions, which may be either round or multilobulated. On the other hand, type II tumours are characterised by the presence of two or more distinct and separate lesions. According to Al-Qattan, type II tumours have been associated with a higher rate of local recurrence [11]. This increased recurrence is likely due to the presence of satellite lesions that remain undetected and therefore are not excised during the initial surgical procedure. In such instances, they may not represent a true recurrence of the original tumour, but rather the progression of a previously unrecognised lesion [1,9,13].

Imaging characteristics have also been suggested as potential factors associated with recurrence, including findings such as bony erosion on radiographs and the presence of osteoarthritic changes. Bone erosion identified on plain radiographs has been proposed as a potential reason for recurrence by Reilly et al. (1999) [8]. It reported that 26% of patients with recurrent tumours exhibited osseous pressure erosion, compared to only 9% in those without recurrence. In contrast, Kitagawa et al. (2004) and Lowyck and De Smet (2006) did not find evidence to support a direct association between bone erosion and recurrence [12,14]. Kitagawa et al. argued that the observed bony changes were not indicative of true invasion, but rather represented pressure effect erosion from the adjacent tumour mass. Uriburu and Levy reported a series of 15 cases in which osseous invasion by the tumour was identified. The lesion was surgically removed from the bone, followed by curettage of the resulting osseous cavity. The recurrence rate of these cases was 13% (n = 2) [15].

In our case series, no specific imaging feature was identified in the available pre-operative radiographs or imaging reports that could be linked to recurrence. However, it is important to note that the majority of patients did not undergo standard radiography, and 45% of patients did not have any imaging report available, limiting our ability to assess this hypothesis. In our current clinical practice, US is typically the first-line imaging modality due to its accessibility, while MRI is usually reserved for larger lesions or when US findings are inconclusive or ambiguous. The absence of imaging documentation in nearly half of our cohort further limited our ability to evaluate radiographic predictors of recurrence, and this missing data should be considered when interpreting our negative findings.

Tumour location in the thumb interphalangeal joint and the distal interphalangeal joints of the fingers has also been associated with an increased risk of recurrence [8,9,11]. Di Grazia et al. proposed that this association may be secondary to the specific anatomical characteristics of these regions. In particular, the close proximity to the neurovascular bundle and the limited soft tissue coverage may hinder complete surgical excision, rather than the location itself being an independent risk factor [3]. Our findings are consistent with this proposed association, where a significant link between recurrence and articular location was found. It reinforces the notion that joint involvement may complicate complete tumour resection and potentially contribute to residual tumour tissue.

Kotwal et al. conducted a prospective study involving 48 patients to evaluate the effectiveness of postoperative radiotherapy in reducing recurrence rates. In this study, 14 patients who were considered to be at higher risk of recurrence received adjuvant radiotherapy, either due to suspected incomplete excision or the presence of mitotic figures on histological analysis. The treatment consisted of a total dose of 20 Gy, administered in daily doses of 2 Gy. None of the patients in the radiotherapy group experienced a recurrence, and two cases were observed among the patients who did not receive postoperative radiotherapy [2]. In our series, radiotherapy was not considered, as our work was a retrospective review of surgical cases only. The study by Kotwal et al. was conducted in an experimental setting, and adjuvant radiotherapy has not been adopted in our clinical practice for the management of GCT-TS.

This study has several limitations. First, its retrospective design introduces potential biases in data completeness and consistency. Second, the sample size was relatively small (n = 90), which limits the statistical power and the generalisation of the findings. Additionally, the marked imbalance in the outcome variable (only 10% recurrence) raises concerns about model reliability and can lead to biased estimates and increase the risk of missing real associations due to insufficient statistical power. Although Firth’s penalised likelihood method was used to mitigate this, it is still sensitive to extreme imbalances and sparse data.

One important limitation lies in the reliance on telephone follow-up as the main tool for recurrence detection, which may have underestimated the true recurrence rate by missing subclinical or asymptomatic lesions. The lack of standardised functional outcome measures or validated quality-of-life questionnaires restricts our ability to assess long-term patient-reported outcomes and functional recovery in a systematic way.

Recurrence-free survival analysis was not performed in our cohort. Establishing the precise time of recurrence proved challenging, as some patients did not seek medical consultation immediately after noticing symptoms, and in clinically suspected cases without histological confirmation, the onset of recurrence was even harder to define. For this reason, we reported recurrence as proportions rather than time-to-event estimates.

Finally, the long study period may have included changes in surgical technique, imaging quality, or follow-up protocols that could have influenced outcomes.

## Conclusions

Although numerous studies have aimed to identify potential risk factors for recurrence, the only widely accepted factor among most authors remains incomplete surgical excision. To minimise this risk, thorough pre-operative imaging is essential in order to identify possible satellite lesions or anatomical characteristics that could complicate complete removal. Our results further suggest that lesions located at finger joints are significantly more likely to recur, underlining the importance of meticulous surgical planning in these cases.

A careful surgical technique is crucial for its complete excision. While recurrences of these tumours are benign in nature, achieving complete removal in a single procedure contributes to a more favourable patient experience and optimises clinical outcomes by reducing the need for further surgeries. However, given the retrospective design, reliance on telephone follow-up for recurrence detection, and absence of standardised functional outcome measures, our findings should be interpreted with caution. Prospective studies with systematic imaging follow-up and validated functional assessments are needed to confirm these results.
